# Impaired Cognitive Function and Altered Hippocampal Synaptic Plasticity in Mice Lacking Dermatan Sulfotransferase Chst14/D4st1

**DOI:** 10.3389/fnmol.2019.00026

**Published:** 2019-02-11

**Authors:** Qifa Li, Xuefei Wu, Xueyan Na, Biying Ge, Qiong Wu, Xuewen Guo, Michael Ntim, Yue Zhang, Yiping Sun, Jinyi Yang, Zhicheng Xiao, Jie Zhao, Shao Li

**Affiliations:** ^1^Liaoning Provincial Key Laboratory of Cerebral Diseases, Department of Physiology, Dalian Medical University, Dalian, China; ^2^National-Local Joint Engineering Research Center for Drug-Research and Development (R & D) of Neurodegenerative Diseases, Dalian Medical University, Dalian, China; ^3^Department of Urology, Dalian Friendship Hospital, Dalian, China; ^4^Department of Anatomy and Developmental Biology, Monash University, Melbourne, VIC, Australia

**Keywords:** dermatan sulfate, Chst14/D4st1, synaptic plasticity, learning and memory, LTP

## Abstract

Chondroitin sulfate (CS) and dermatan sulfate (DS) proteoglycans (PGs) are major extracellular matrix (ECM) components of the central nervous system (CNS). A large body of evidence has shown that CSPGs/DSPGs play critical roles in neuronal growth, axon guidance, and plasticity in the developing and mature CNS. It has been proposed that these PGs exert their function through specific interaction of CS/DS chains with its binding partners in a manner that depends on the sulfation patterns of CS/DS. It has been reported that dermatan 4-O-sulfotransferase-1 (Chst14/D4st1) specific for DS, but not chondroitin 4-O-sulfotransferase-1 (Chst11/C4st1) specific for CS, regulates proliferation and neurogenesis of neural stem cells (NSCs), indicating that CS and DS play distinct roles in the self-renewal and differentiation of NSCs. However, it remains unknown whether specific sulfation profiles of DS has any effect on CNS plasticity. In the present study, Chst14/D4st1-deficient (*Chst14*^−/−^) mice was employed to investigate the involvement of DS in synaptic plasticity. First, behavior study using Morris Water Maze (MWM) showed that the spatial learning and memory of *Chst14*^−/−^ mice was impaired when compared to their wild type (WT) littermates. Corroborating the behavior result, long-term potentiation (LTP) at the hippocampal CA3-CA1 connection was reduced in *Chst14*^−/−^ mice compared to the WT mice. Finally, the protein levels of N-Methyl-D-aspartate (NMDA) receptor, α-amino-3-hydroxy-5-methyl-4-isoxazolepropionic acid (AMPA) receptor, postsynaptic density 95 (PSD95), growth associated protein 43 (GAP-43), synaptophysin (SYN) and N-ethylmaleimide sensitive factor (NSF) which are important in synaptic plasticity were examined and Chst14/D4st1 deficiency was shown to significantly reduce the expression of these proteins in the hippocampus. Further studies revealed that Akt/mammalian target rapamycin (mTOR) pathway proteins, including protein kinase B (p-Akt), p-mTOR and p-S6, were significantly lower in *Chst14*^−/−^ mice, which might contribute to the decreased protein expression. Together, this study reveals that specific sulfation of DS is critical in synaptic plasticity of the hippocampus and learning and memory, which might be associated with the changes in the expression of glutamate receptors and other synaptic proteins though Akt/mTOR pathway.

## Introduction

Proteoglycans (PGs) are important components of the extracellular matrix (ECM) formed by covalent attachments of glycosaminoglycans (GAGs) to serine residues of core proteins (Bian et al., 2011). GAGs are linear polysaccharides consisting of repeated disaccharide units which can be sulfated at different positions to create a vast structural microheterogeneity of chains with different function, for instance, dermatan sulfate (DS), chondroitin sulfate (CS) and heparan sulfate (HS). PGs are known to contribute to normal embryonic and postnatal development and tissue homoeostasis by ensuring tissue stability and signaling functions, such as cell migration, proliferation and survival. GAGs can be classified into two types, one is galactosaminoglycans with CS and DS, the other one is glucosaminoglycans represented by HS (Dündar et al., 2009; Krichen et al., 2017; Ramachandra et al., 2017; Soares da Costa et al., 2017).

CSPG/DSPG are major ECM components of the central nervous system (CNS) and have the potential to interact with a wide range of growth factors and neurotrophic factors that influence neuronal migration, axon guidance, neurite outgrowth and synaptic plasticity (Miller and Hsieh-Wilson, 2015; Miyata and Kitagawa, 2015). It has been proposed that CSPG/DSPG exert their function through specific interaction of CS/DS chains with its binding partners in a manner that depends on the sulfation patterns of CS/DS, e.g., the participation of CS/DS in neurosphere formation (Von Holst et al., 2006). ChondroitinaseABC (ChaseABC), which unselectively degrades CS and DS and has been used in a lot of studies to investigate the function of CS/DS. Thus, it is difficult to discern the function of DS and CS which might actually be different. Treatment of neural stem cells (NSCs) from the embryonic mouse telencephalon with ChaseABC resulted in diminished proliferation and impaired neuronal differentiation of NSCs (Sirko et al., 2007). However, dermatan 4-O-sulfotransferase-1 (Chst14/D4st1) that is specific for DS, but not chondroitin 4-O-sulfotransferase-1 (Chst11/C4st1) specific for CS, regulates proliferation and neurogenesis of NSCs (Bian et al., 2011). This indicates that CS and DS play distinct roles in the self-renewal and differentiation of NSCs. It is still unclear whether specific sulfation profiles of DS has any effect on CNS plasticity.

In terms of the structure, DS is a copolymer which consists of alternating disaccharide units of l-iduronic acid (IdoUA) and N-acetyl-d-galactosamine (GalNAc) with 50–200 repeats (Mizumoto et al., 2017). DS chains can be sulfated at the hydroxy groups of C-2 on IdoUA and the C-4 positions of GalNAc residues by various sulfotransferases. There are three major sulfotransferases which take part in this process and have different substrate specificities (Mitsunaga et al., 2006). C4st1 preferably sulfates a GalNAc flanked by two GlcA residues while Chst14/D4st1 prefers two flanking IdoA residues. C4st2 can equally sulfate both substrates (Pacheco et al., 2009). Among the three sulfotransferases, Chst14/D4st1 is a key and specific enzyme that cannot be replaced by other sulfotransferases in the process of synthesizing DSs (Bian et al., 2011). Chst14/D4st1-deficient (*Chst14*^−/−^) mice are useful for studying the functions of specific sulfation profile of DS.

In the current study, we investigated the role of DS sulfation in synaptic plasticity as well as learning and memory using *Chst14*^−/−^ mice. Our data showed that Chst14/D4st1 deficiency resulted in impaired spatial learning and memory as well as long-term potentiation (LTP). We also found that the protein levels of α-amino-3-hydroxy-5-methyl-4-isoxazolepropionic acid (AMPA) receptor subunit GluA1, N-Methyl-D-Aspartate (NMDA) receptor subunit NR2B, postsynaptic density protein 95 (PSD95), growth-associated protein 43 (GAP-43), synaptophysin (SYN) and N-ethylmaleimide sensitive factor (NSF) protein kinase B (p-Akt) and p-S6 were decreased in *Chst14*^−/−^ mice. Our results suggest that specific sulfation profile of DS is indispensable for synaptic plasticity that might be associated with downregulation of synaptic proteins though Akt/mammalian target rapamycin (mTOR) signaling pathway.

## Materials and Methods

### Animals

Mice (C57BL/6J) were kept in the conventional housing unit under standard conditions (five per cage, 24°C, 45%–65% humidity, 12 h light/dark cycle), with free accessing to food and water. This study was carried out in accordance with the recommendations of National Institute of Health Guide for the Care and Use of Laboratory Animals (NIH Publications No. 80-23) revised 1996. All experimental protocols were approved by the Institutional Ethics Committee of the Dalian Medical University, and all efforts were made to minimize the number of animals used and their suffering.

### Morris Water Maze

The protocol used was described by Morris (1984). The Morris Water Maze (MWM) apparatus consisted of a tank which is 120 cm in diameter, 60 cm in height and was divided into four quadrants. The tank was filled with water (temperature, 25 ± 1°C) until the platform (10 cm in diameter) was submerged 1 cm below the water. Four visual cues were placed on the walls of the tank (in each quadrants) as spatial references for mice to determine their navigation path. Above the center of the pool, a camera was used to detect the position of the animals and Ethosvision software was used to record the real time data. Before the experiment, the animals (3–4 months old) were placed in the pool without any platform for 30 s to let the animals get familiar with the environment. In the hidden platform acquisition test, animals were trained four trials per day for five consecutive days. The starting positions were done in the quadrants and alternated on each trial. The duration of a trial was 90 s, after which mouse was manually guided to the platform and allowed to stay on it for 10 s if it could not find it. Otherwise, it was allowed to remain on the platform for 10 s before the next trial. One day after the last trial, we removed the platform and performed the probe test. The escape latency and path length were measured, and the numbers of platform-site crossovers were recorded. The results are expressed as mean ± standard error of the mean (SEM).

### Electrophysiological Recordings

Hippocampi were dissected from the brain of 3–4 months old mice and acute 300 μm thick slices were prepared using a vibratome (LEICA VT1200S) in cold artificial cerebrospinal fluid (ACSF, 4°C) bubbled with 95% O_2_ and 5% CO_2_ containing: 110 mM NaCl, 2.5 mM KCl, 1.5 mM MgSO_4_·2H_2_O, 2.5 mM CaCl_2_, 1.25 mM NaH_2_PO_4_, 26 mM NaHCO_3_ and 10 mM D-glucose (pH 7.3). Prior to stimulation, hippocampal slices were maintained at room temperature in ACSF for at least 1 h before being removed to a submersion-recording chamber and was continually perfused with oxygenated ACSF at the rate of 1–2 mL per minute. Test stimuli were delivered at 0.033 Hz (0.2 ms duration) through concentric bipolar electrodes, placed in the CA1 area of the hippocampal slice to stimulate Schaffer Collateral (SC) pathway. Field excitatory postsynaptic potentials (fEPSPs) were recorded from the stratum radiatum of CA1 using glass microelectrodes filled with 3 M NaCl solution (resistance 2–5 MΩ). After baseline was recorded for 30 min at an intensity that was set to 40%–50% of the maximal response, LTP was induced using high frequency stimulation (four 100 Hz and 1 s trains delivered 20 s apart).

The data were acquired with an Axon multiclamp 700 B amplifier, filtered at 0.1e5 KHz, and digitized at 10 KHz, and analyzed offline by pClamp10.3 software (Molecular Devices Corp, USA).

Input-output (I/O) curves were established by single-pulse stimulation of the SCs region in order to evaluate synaptic efficacy by adjusting the stimulus intensity between by steps 0.05–1.0 mA. Stimulus pulses were delivered at 0.033 Hz and five responses at each current intensity were averaged.

Presynaptic function was explored by using paired-pulse facilitation (PPF) paradigm with inters-stimulus intervals (ISIs) ranging from 25, 50, 75, 100, 125, 150 and 200 ms. Facilitation was measured as a ratio of the second pulse-evoked EPSP slope to the first evoked, averaged over five responses per pulse pair.

### Western Blot

Hippocampi were homogenized in RIPA buffer with 1% cocktail. The lysates were centrifuged at 12,000 rpm for 30 min at 4°C and the supernatants were collected. Proteins were separated by 10% SDS-PAGE gel and transferred onto PVDF membranes (Millipore, Billerica, MA, USA). Then the membranes were blocked in 5% nonfat dried milk for 1 h at room temperature and incubated overnight at 4°C with primary antibodies: GluA1 (1:1,000, Abcam, ab31232), NR1 (1:500, BD Pharmingen, 556308), NR2A (1:500, Millipore, MAB5216), NR2B (1:1,000, Abcam, ab93610), PSD95 (1:1,000, Abcam, ab2723), GAP-43 (1:1,000, Millipore, AB5220), NSF (1:500, Cell Signaling Technology, 2145S), SYN (1:1,000, Millipore, MAB5258-I), p-S6 (1:1,000, Cell Signaling Technology, 4858), p-Akt (1:1,000, Cell Signaling Technology, 4060), p-mTOR (1:1,000, Cell Signaling Technology, 5536), actin (1:2,000, Abcam, ab6276). After being washed three times with TBST, the membranes were incubated with the HRP-conjugated secondary antibodies for 1 h at room temperature, and then detected by enhanced chemiluminescence (ECL, Biotool). Protein bands were quantified and analyzed with ImageJ. For some proteins including GluA1, NR1, GAP-43, PSD95 and β-actin, immunoblots were performed on the different parts of PVDF membrane from the same gel, thus their bands were normalized to the same β-actin bands, although the protein levels were expressed separately in results, which is the case of NR1, GluA1, PSD95 and GAP-43 in Figure 3.

**Figure 1:**
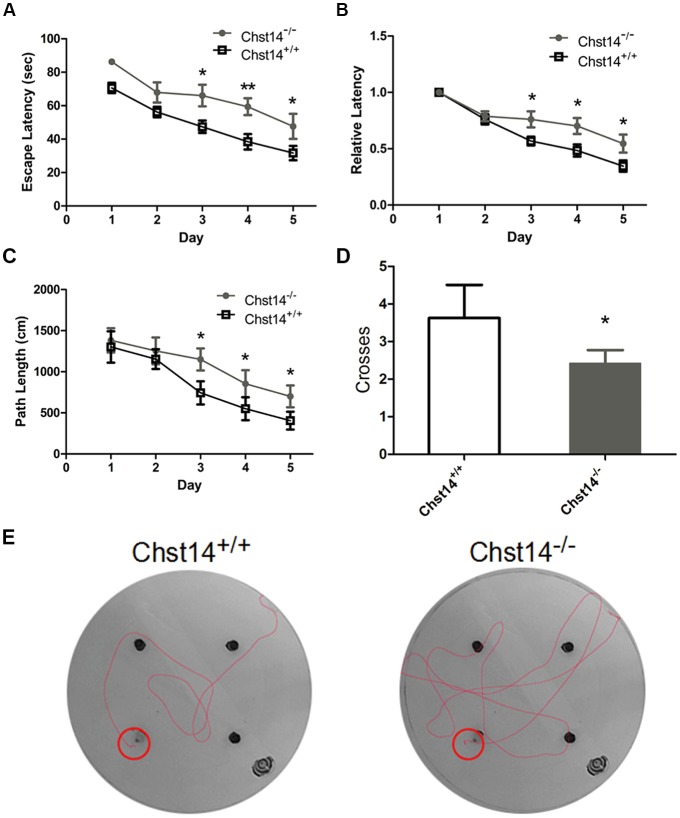
Chst14/D4st1 deficiency leads to impaired spatial learning and memory. Morris Water Maze (MWM) tests were performed on wild type (WT; *n* = 16) or *Chst14*^−/−^ mice (*n* = 12) and animal behaviors were recorded and analyzed. **(A)** The escape latencies in each group of the mice were analyzed.** (B)** The escape latencies of each group of mice on the first day were normalized to 1.0. The relative escape latencies in the subsequent days to that of the first day were calculated. **(C)** The average distances that the mice spent to find the platform. **(D)** The times that each group of mice swam across the target sites after retrieval of the platform. **(E)** Representative images of the path that the mice swam along to find the platform. Data are presented as mean ± standard error of the mean (SEM) in each group. *p < 0.05, **p < 0.01.

**Figure 2:**
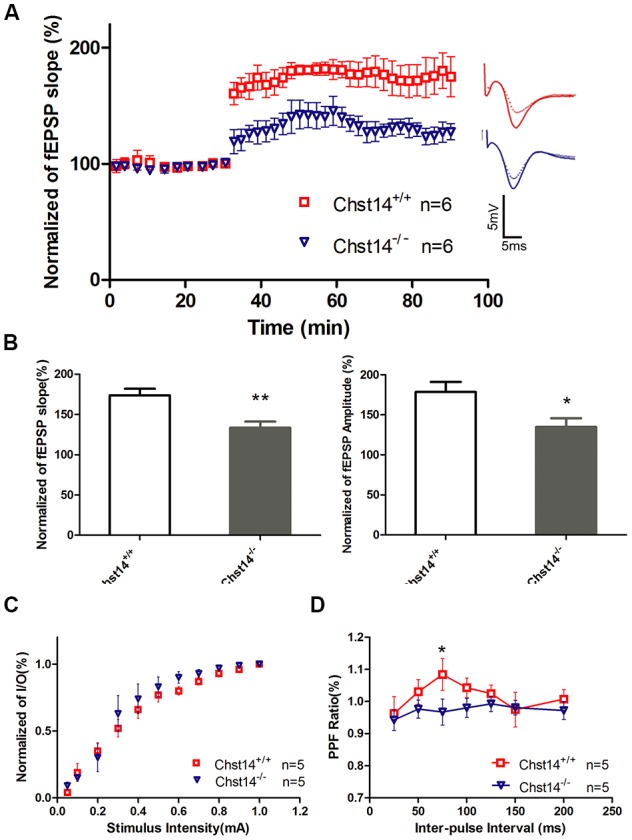
Chst14/D4st1 deficiency reduces long-term potentiation (LTP) formation in hippocampal slices. Hippocampal slices from WT or *Chst14*^−/−^ mice at 3 months of age (*n* = 6 mice for each group) were freshly prepared and subjected to LTP induction and analysis. **(A)** Time course of the effects of high-frequency stimulation (HFS) on the field excitatory postsynaptic potential (fEPSP) initial slope. **(B)** Cumulative data showing the mean fEPSP peak amplitude and the mean fEPSP slope 60 min post-HFS. **(C)** Input-output (I/O) plots of fEPSP slopes vs. current input (mA) were similar in WT and *Chst14*^−/−^ mice (five slices from four mice), indicating that lack of dermatan sulfate (DS) does not alter baseline synaptic transmission. **(D)** Paired pulse facilitation (PPF) analysis showing the S2/S1 ratios for increasing stimulation interpulse intervals in slices from WT and *Chst14*^−/−^ mice (five slices from four mice). Error bars represent SEM. *p < 0.05, **p < 0.01.

**Figure 3:**
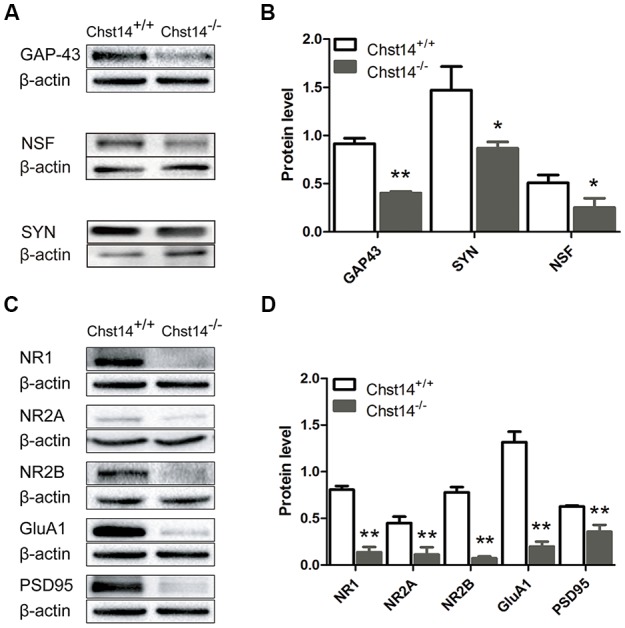
Chst14/D4st1 deficiency decreases protein expression of the synaptic proteins in the hippocampus. Total proteins from WT or *Chst14*^−/−^ mice hippocampi were subjected to Western blot analysis to determine the protein levels of the synaptic proteins. **(A,B)** Representative immunoblots and densitometric analysis of the immunoblots showed that the expression levels of growth associated protein 43 (GAP-43), N-ethylmaleimide sensitive factor (NSF) and synaptophysin (SYN) in the hippocampi were significantly decreased in the *Chst14*^−/−^ mice. **(C,D)** Representative immunoblots and densitometric analysis of the immunoblots showed that the expression levels of NR1, NR2A, NR2B, GluA1 and postsynaptic density 95 (PSD95) were significantly decreased in the *Chst14*^−/−^ mice. Here, NR1, GluA1 and β-actin immunoblots were performed on the different parts of PVDF membrane from the same gel, thus NR1 and GluA1 were normalized to the same β-actin bands. Graphs represent the means ± SEM (*n* = 4 mice for each group). GAP-43, PSD95 and β-actin immunoblots were performed on the different parts of PVDF membrane from the same gel, thus GAP-43 and PSD95 were normalized to the same β-actin bands. Graphs represent the means ± SEM (*n* = 4 mice for each group). *p < 0.05, **p < 0.01.

### Statistical Analyses

All statistical analyses were performed using SPSS18.0. Data are presented as the mean ± SEM. Data between multiple groups were analyzed by one- or two-way analysis of variance followed by Fischer protected least significant difference *post hoc* tests. Unpaired *t-test* was used to analyze differences between two groups. *p* Value < 0.05 was considered as the significance level for all analyses (*p < 0.05, **p < 0.01).

## Results

### *Chst14*^−/−^ Mice Show Deficits in Spatial Learning and Memory

As DS plays an important role in self-renewal and differentiation of NSCs, we investigated whether DS has any effect on learning and memory using the MWM test. In the test, mice were trained for 5 days with a hidden platform. During each trial, the escape latency was measured as an index of the spatial learning ability. During the first 2 days, we found no significant differences between the two groups. However, *Chst14*^−/−^ mice showed much higher escape latencies during 3–5 days of training trial compared to wild type (WT) mice (Figure 1A). To eliminate the influence of swimming speed, we normalized the escape latencies in the first trial of each group to 1.0. The relative escape latencies in the subsequent trial days were then quantified to that in the first trial (Figure 1B). This could enable us to compare the spatial learning ability of each group taking into consideration their differences in swimming speed. As shown in Figure 1B, compared with WT mice, *Chst14*^−/−^ mice failed to show a learning trend indicated by the shortening escape latencies as days of training passed. Consistently, the swimming length of *Chst14*^−/−^ mice was increased compared with WT mice (Figure 1C). In the probe test, the number of platform crossing were measured to evaluate spatial memory ability. *Chst14*^−/−^ mice showed a significantly decreased number of platform crossing (Figure 1D). These results demonstrate that Chst14 deletion results in impaired learning and memory.

### *Chst14*^−/−^ Mice Manifest Impaired LTP

It is well known that the hippocampus plays important roles in long-term memory (Luo et al., 2011). Synaptic loss and synaptic abnormalities are strongly correlated with cognitive impairment. LTP is a very important form of synaptic plasticity and is the most extensively studied cellular model for learning and memory (Petrovic et al., 2017). To investigate whether the behavioral deficits in *Chst14*^−/−^ mice were associated with altered hippocampal synaptic plasticity, LTP was induced by high-frequency stimulation (HFS; four 100 Hz and 1 s trains were delivered 20 s apart) at SC-CA1 synapses in hippocampal slices of 3-month-old *Chst14*^−/−^ and WT mice (Zhao et al., 2015). Figure 2A shows the changes in fEPSPs slope before and after HFS in different mice. As shown in the Figure 2B, LTP was significantly attenuated in *Chst14*^−/−^ mice, as indicated by the decreased fEPSP slope (173.75% ± 8.12% vs. 133.41% ± 7.66%) and amplitude (178.50% ± 12.56% vs. 134.76% ± 11.029%) compared with WT mice. There was no different between WT and *Chst14*^−/−^ mice on baseline responses in a control pathway (Supplementary Figure S1). This is consistent with the observation in learning and memory in *Chst14*^−/−^ mice. These data confirm that Chst14 plays an important role in synaptic plasticity. To examine whether the properties of basic synaptic transmission at SC-CA1 synapses is altered in *Chst14*^−/−^ mice, input-output curves were obtained by measuring the post-synaptic potential slope with varying stimulus intensities (0.05–1.0 mA). Data were normalized using 100% as the highest amplitude (average of five selected sweeps in each stimulation intensity) of the fEPSP. *Chst14*^−/−^ mice showed no significant change in the input-output curve compared to WT littermates (Figure 2C). This indicates that DS does not affect the basal synaptic response. PPF was obtained after the I/O curve measurements to determine the probability of synaptic vesicle release, and this was measured by paired pulses at intervals between 25 and 200 ms. As shown in Figure 2D, the basal PPF at a 75-ms inter-stimulus interval was significantly decreased in *Chst14*^−/−^ mice compared WT littermates. This finding suggests that DS may interfere with probability of synaptic vesicle release from the pre-synaptic terminals.

### The Synaptic Proteins Are Decreased in *Chst14*^−/−^ Mice

It is known that both presynaptic and postsynaptic mechanisms are involved in LTP (Nicoll and Malenka, 1995). As synaptic proteins are critical for synaptic transmission, it is of interest to see whether Chst14/D4st1 deficiency has any effect on expressions of proteins that are involved in synaptic plasticity.

GAP-43 is neuron-specific and found in high concentrations in growth cones. It plays an important role in the process of learning and memory (Li et al., 2016; Moghimi et al., 2016). As a marker of synaptic activity, SYN is the main membrane protein of presynaptic vesicles involved in vesicle formation and exocytosis (Valtorta et al., 2004). Meanwhile, the function of NSF is essential for a highly dynamic response before synaptic vesicles fuse with presynaptic plasma membranes to release neurotransmitters (Kuner et al., 2008). The results show that GAP-43, NSF and SYN were decreased in the hippocampus of *Chst14*^−/−^ mice (Figures 3A,B), supporting dysfunction of presynaptic membrane.

For postsynaptic proteins, we first examined NMDA and AMPA receptors that play major roles in hippocampus-dependent learning and memory as well as LTP (Cull-Candy et al., 2001; Tu and Kuo, 2015). NMDA receptors are heterotetramer formed by two glycine-binding NR1 subunits and two glutamate-binding NR2 subunits (NR2A, NR2B, NR2C and NR2D). NR2A or NR2B combined with NR1 are the major forms of NMDA receptors and play important roles in synaptic plasticity in the adult brain (Sachser et al., 2017). Accordingly, we determined the protein expression of NMDA subunits NR1, NR2A and NR2B in the hippocampus of WT and *Chst14*^−/−^ mice. The results show that the protein expression levels of NR1, NR2A and NR2B (Figure 3) were decreased in the hippocampus of *Chst14*^−/−^ mice compared with WT mice. AMPA receptors are heterotetramers composed of various subunits (GluA1–4) usually permeable to Na^+^ and K^+^ (Sachser et al., 2017). They are widely expressed in the brain with different functions. During the induction of LTP, the recruitment of GluA1-containing AMPA receptors to the post-synaptic membrane is a critical step (Panja and Bramham, 2014). Hence, we also measured the protein expression levels of GluA1 from the hippocampus of WT and *Chst14*^−/−^ mice (Figures 3C,D). It was significantly decreased in the hippocampus of *Chst14*^−/−^ mice compared with WT mice. PSD95 plays a key role to determine the PSD size and synaptic strength in neuronal development, experience-dependent plasticity, like LTP and LTD (Holahan et al., 2007). In this study, it was shown that the protein expression level of PSD95 was decreased significantly in the hippocampus of *Chst14*^−/−^ mice compared with WT mice (Figures 3C,D). These results suggested that DS may affect synaptic transmission through regulating the expression of postsynaptic proteins in the hippocampus.

### The Protein Levels of the Akt/mTOR Signaling Pathway Are Decreased in *Chst14*^−/−^ Mice

**Figure 4:**
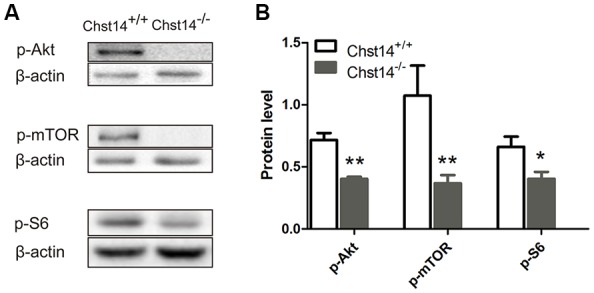
Chst14/D4st1 deficiency reduces the protein expression of Akt/mammalian target rapamycin (mTOR) signaling pathway in the hippocampus. Total proteins from WT or *Chst14*^−/−^ mice hippocampi were subjected to Western blot analysis to determine the protein levels of p-Akt, p-mTOR and p-S6. **(A,B)** Representative immunoblots and densitometric analysis of the immunoblots showed that the expression levels of p-Akt, p-mTOR and p-S6 in the hippocampi were significantly decreased in the *Chst14*^−/−^ mice. *p < 0.05, **p < 0.01.

The above results have shown significant down-regulation of both presynaptic and postsynaptic proteins, we then tried to explore the signaling pathways of DS affecting protein expression. In previous reports, the Akt/mTOR pathway plays an important role in protein synthesis (Chen et al., 2016). Ribosomal protein S6 is essential for protein translation and is acutely phosphorylated by S6K, an established downstream target of mTOR (Sawicka et al., 2016). Our results showed that p-Akt, p-mTOR and p-S6 were decreased in the *Chst14*^−/−^ mice (Figure 4), suggesting that Akt/mTOR pathway may contribute to decreased protein synthesis in *Chst14*^−/−^ mice. There was no difference between trained and untrained animals (Supplementary Figure S2).

## Discussion

As DS-specific sulfotransferase Chst14 regulates proliferation and neurogenesis of NSCs (Bian et al., 2011), it is rational to see whether Chst14 plays a role in the adult CNS function such as synaptic plasticity. In the present study, we investigated the changes of spatial learning/memory and LTP as well as expression of several proteins that are associated with synaptic plasticity in *Chst14*^−/−^ mice.

In order to study whether Chst14/D4st1 deficiency affects learning and memory, MWM test was used in the present study to evaluate spatial learning and memory ability in mice through the training trial and probe trial. When animals have problems in finding the platform in the water maze, it indicates their inability to remember the spatial information which was supposed to have been acquired during the training days, reflecting deficits in hippocampal-dependent spatial cognition (Kim et al., 2016). In our study, it was shown that the spatial learning and memory was impaired in *Chst14*^−/−^ mice (Figure 1). Synaptic plasticity is considered the basis of learning and memory (Gu et al., 2016). As a physiological pattern of synaptic plasticity, LTP is correlated with hippocampal-dependent memory (Yang et al., 2017). Here, LTP from SCs to hippocampal CA1 region was measured after MWM test. Our results indicated that LTP induction was dampened in *Chst14*^−/−^ mice (Figures 2A,B), which probably could explain their performance in the behavioral test. In order to assess the differences that potentially exist in baseline synaptic responsiveness, the I/O function was measured in this study. However, we noted there was no difference in synaptic transmission at baseline between WT and *Chst14*^−/−^ mice (Figure 2C). PPF is a phenomenon of short-term plasticity whereby a second synaptic response is enhanced by a preceding stimulation of similar intensity. This phenomenon determines the probability of vesicle release and is usually increased due to increased calcium entry into presynaptic terminals (Shang et al., 2016). In our data, we report of decrease in PPF function in *Chst14*^−/−^ mice which may indicate that the probability of presynaptic glutamate release was decreased in *Chst14*^−/−^ mice.

As changes in synaptic proteins or receptors associated with learning and memory may be the structural basis for the defect in synaptic functions, we examined the levels of these proteins in presynaptic and postsynaptic membranes, respectively. GAP-43, a nervous system-specific protein enriched at presynaptic nerve terminals, is thought to be involved in axonal outgrowth and plasticity in synaptic connections (Kristjansson et al., 1982). It can be phosphorylated by protein kinase C (PKC; De Graan et al., 1990) and its phosphorylation level is directly related to LTP and learning and memory (Gianotti et al., 1992; Young et al., 2000). In GAP-43 heterozygous knockdown mice, hippocampal-dependent memory is reported to be impaired (Rekart et al., 2005). Overexpression of GAP-43 has been shown to increase LTP in dentate gyrus and improve learning in an 8-arm radial maze (Routtenberg et al., 2000). Involvement of GAP-43 in learning and memory has been proposed in two ways. First, GAP-43 is located in the presynaptic terminal and can directly interact with components that regulate the release of neurotransmitters including soluble NSF attachment protein (SNARE) complex proteins to modulate presynaptic neurotransmitter release (Haruta et al., 1997). Second, PKC in presynaptic terminal can be activated by a NMDA-dependent postsynaptic retrograde signal. The phosphorylated GAP-43 then interacts with calcium-sensing proteins of the EPM (exocytotic protein machine) to enhance the release of neurotransmitters when intraterminal calcium is raised sufficiently (Routtenberg et al., 2000). NSF plays a key role in eukaryotic trafficking and is essential for maintaining pools of fusion-ready individual SNARE proteins that mediate membrane fusion in a variety of cellular processes, including neurotransmitter release, protein transport, and hormone secretion (Zhao and Brunger, 2016). As one of the most abundant synaptic vesicle proteins, SYN can interact with synaptobrevin which is a key SNARE protein and is involved in neurotransmitter release (Egbujo et al., 2016). Hence, the decrease in protein expression levels of GAP-43, NSF and SYN in* Chst14^−/−^* mice (Figures 3A,B) might result in weakened release of presynaptic neurotransmitters followed by affected learning and memory.

Glutamate receptors are the most important receptors for excitatory amino acids in CNS. They have been shown to be crucial for the formation of synapses, synaptic plasticity as well as learning and memory (Yan et al., 2014). NMDA and AMPA receptors, two important ionotropic glutamate receptors, have been proven to participate in regulating many important functions in the CNS such as LTP and the development of neural plasticity (Cull-Candy et al., 2001; Tu and Kuo, 2015). Extensive research effort including gene knockout, agonists and antagonists have been used in identifying the roles of NMDA/AMPA receptors in LTP. For instance, the NMDA receptor antagonist (2R)-amino-5-phosphonovaleric acid (APV) has been reported to block LTP induction (Bourne et al., 2013). NR2B-overexpressing mice show increased LTP (Cui et al., 2011). In adult GluA1^−/−^ mice, the induction of LTP failed (Zamanillo et al., 1999). During the initial phase of LTPGluA2-lacking AMPA receptors increase at CA1 SC synapses through an insertion from the intracellular pools (Rozov et al., 2012). Thus, alterations in the expression of hippocampal NMDA and AMPA receptors have been proposed to impact synaptic plasticity and learning and memory. Our results showed that Chst14 deficiency led to a strong reduction in the hippocampal expression of the NMDA subunit NR1, NR2A, NR2B and the AMPA subunit GluA1 (Figures 3C,D).

In addition to these receptors in the post-synaptic membrane, we also checked the expression of PSD95. PSD95 is highly enriched in the PSD and is the most widely studied in synaptic plasticity among the four PSD-MAGUKs (PSD95-like membrane associated guanylate kinases) family members (Chen et al., 2005). It interacts with the subunits of NMDA/AMPA receptors to affect the stability of these receptors and their participation in synaptic plasticity. During early development of the brain, NR2B-to NR2A-subunit switch can be found in most regions and can promote synaptic maturation (Dumas, 2005). In this process, PSD95/NR2A complexes do replace synapse-associated protein 102 (SAP102)/NR2B complexes indicating PSD95 is a developmental regulator of NMDA receptor (Coley and Gao, 2018). In the hippocampus of PSD95^−/−^ mice, the protein level of GluA1 is significantly decreased (Béïque et al., 2006), prompting that the downregulation of PSD95 affects synaptic function. Furthermore, overexpressing PSD95 in hippocampal neurons causes an increase of AMPA receptor and dendritic spine density (El-Husseini et al., 2000). In *Chst14*^−/−^ mice, downregulation of PSD95 may attenuate the regulation of PSD95 on NMDA/AMPA receptors, affecting receptor function and synapse development.

Together, our study suggests that specific sulfation profile of DS promotes the synaptic plasticity in the hippocampus and enhances spatial learning and memory. Both presynaptic and postsynaptic changes in protein expression might contribute to the synaptic defect caused by Chst14/D4st1 deficiency. Since Chst14 deficiency does not affect the volume and thickness of the motor cortex, the volume of CA1/dentate gyrus regions of hippocampus and the density of neurons and astrocytes in these brain regions (Bian et al., 2011), the reduction of synaptic proteins observed here is probably due to regulation on protein expression by DSPG, but not the consequence of reduced number of mature neurons.

Previous reports have shown that PGs can participate in learning and memory in different ways. Biglycan, a neurotrophic brain-derived CS proteoglycan, was found to facilitate learning when injected into the posterior part of the ventral pallidum (De Souza Silva et al., 2002). Meanwhile CSPG is known to act as major inhibitors of the structural and functional plasticity of neural circuits which might be due to interaction of CS with some growth factors and neurotrophic factors (Miyata and Kitagawa, 2015; Mizumoto et al., 2015; Ohtake et al., 2016). Both Crtl1-deficient mice and chondroitinase ABC-treated WT mice show an enhanced long-term object recognition memory in the perirhinal cortex (Romberg et al., 2013). Compared with CS, DS involves a variety of biological processes due to the existence of IdoUA whose pyranose ring tends to form various conformations, causing interaction with various partners to perform different functions (Nandini et al., 2005). DSPGs can regulate biological processes at or near the cell surface through binding several growth factors, cytokines, chemokines, and adhesion molecules. As the co-receptors of various growth factors, DSPGs are involved in migration and intracellular signal transduction by linking the external environment with intracellular signal transduction (Malmström et al., 2012). For instance, the interaction between DS chain and fibroblast growth factor (FGF)-7 can promote the binding of FGF-7 to a FGF receptor and the cell proliferation (Hashiguchi et al., 2010). Similarly, HS PGs have been suggested to modulate the activities of heparin-binding growth factors (Villena and Brandan, 2004). Previous study also showed that the binding affinity of BDNF to DS was higher than HS (Nandini et al., 2005), suggesting that DS may affect downstream signaling pathway of BDNF after binding to it.

As a member of the neurotrophin family, BDNF is found to be involved in various biological processes in the CNS ranging from neurogenesis to synaptic plasticity and cognition (Guo et al., 2018; Sonal and Raghavan, 2018). The maintenance of hippocampal LTP requires synthesis of new proteins. Local protein synthesis maintains an appropriate level of synaptic strength in cortical and hippocampal neurons, which is related to homeostatic synaptic plasticity (Miller et al., 2014). Binding with its TrkB tyrosine kinase receptor, BDNF stimulates the activation of many signaling pathways, including PI3K/Akt signaling pathway, to promote protein translation. Akt activates mammalian target rapamycin (mTOR), which induces the phosphorylation of p70 ribosomal S6 kinase (p70S6K). Then activated p70S6K induces phosphorylation of small ribosomal protein 6 (S6) whose phosphorylation state correlates with translational rates. mTOR, p70S6K and S6 regulated by Akt pathways are crucial in the regulation of protein translation initiation (García-Gutiérrez et al., 2013). Phosphorylation of S6 leads to increased translation of mRNA such as CaMKIIα, NR1, GluR1, PSD95, synapsin I, all of which have been demonstrated to play core roles in synaptic plasticity (Aakalu et al., 2001; Schratt et al., 2004, 2006).

**Figure 5:**
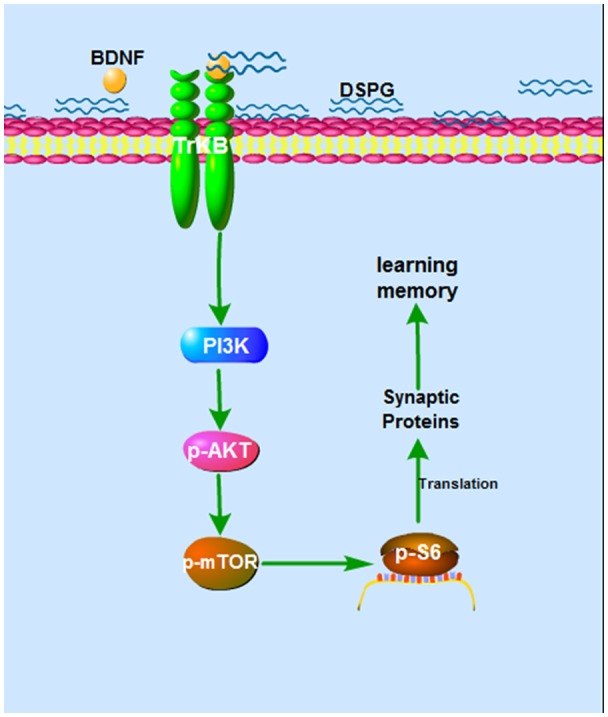
The hypothesized mechanism of DS on learning and memory. Binding of DS to BDNF leads to activation of BDNF activity. The BDNF-TrkB signaling activates Akt pathway, which stimulates mTOR signaling. This can lead, through regulation of p-S6, to increased translation and increased synaptic proteins, which contributes to synaptic plasticity.

In the current study, we examined the protein levels of BDNF-PI3K/Akt-mTOR pathway, showing that p-Akt, p-mTOR and p-S6 were all decreased in the *Chst14*^−/−^ mice (Figure 4), which may contribute at least partially to downregulation of synaptic proteins caused by Chst14/D4st1 deficiency. However, this hypothesis (Figure 5) need to be verified by further investigation, for instance, examining whether activation of this pathway can ameliorate the defect in synaptic function and protein expression of *Chst14*^−/−^ mice.

## Conclusion

In summary, our findings suggest that specific DS sulfation is critical for synaptic plasticity and learning and memory in the hippocampus, which might be associated with regulation on presynaptic and postsynaptic protein expression by DSPG.

## Author Contributions

SL, ZX and JZ contributed to the conception and design of the project. QL, QW, BG, XN, YS and XG contributed to the experiments. QL, YZ and XW analyzed and interpreted the data. QL, XW, MN, JY and SL wrote and revised the manuscript.

## Conflict of Interest Statement

The authors declare that the research was conducted in the absence of any commercial or financial relationships that could be construed as a potential conflict of interest.
