# Supplementary material for: Impaired Cognitive Function and Altered Hippocampal Synaptic Plasticity in Mice Lacking Dermatan Sulfotransferase Chst14/D4st1
Source: Front Mol Neurosci. 2019 Feb 11;12:26. doi: 10.3389/fnmol.2019.00026 (PMC6396735; doi:10.3389/fnmol.2019.00026)
Supplement: Supplementary file 1 [file Data_Sheet_1.doc]

**supplementary materials**

**

**

**Figure.1 Summary of time course of fEPSP slopes in control pathways.**


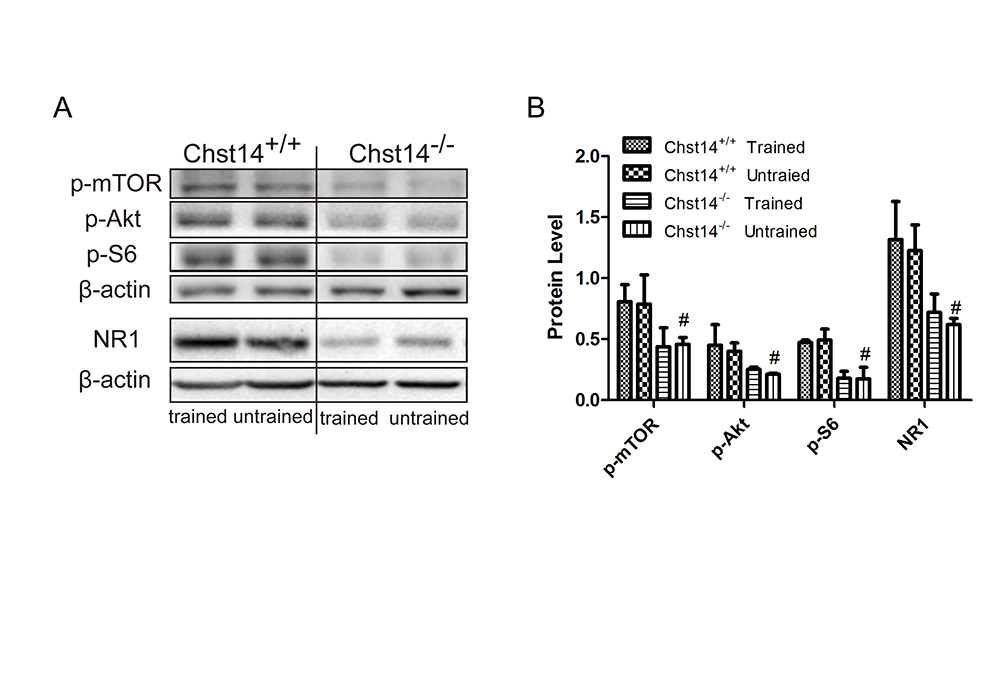


**Figure.2 The protein expression of p-Akt, p-mTOR, p-S6 and NR1in trained and untrained animals.** Total proteins from untrained WT or *Chst14^-/-^* mice hippocampi were subjected to Western blot analysis to determine the protein levels of p-Akt, p-mTOR, p-S6 and NR1. **(A-B)** Representative immunoblots and densitometric analysis of the immunoblots showed that the expression levels of p-Akt, p-mTOR, p-S6 and NR1 in the hippocampi were significantly decreased in the untrained *Chst14^-/-^* mice.^#^ *p* < 0.05.
